# Canine rabies vaccination, surveillance and public awareness programme in Beijing, China, 2014–2024

**DOI:** 10.2471/BLT.24.291497

**Published:** 2025-01-30

**Authors:** Qi Yu, Jiyu Liu, Haojun Zhao, Huiling Chen, Yuxuan Xiang, Qingbin Liu, Li Mei, Wei Zhang, Minheng Cheng, Zhen Li, Runian Bai, Linlin Zhu, Liuqing Zhang, Songli Li

**Affiliations:** a Beijing Animal Disease Control Center, Beijing, China.; b Institute of Animal Science, Chinese Academy of Agricultural Sciences, 2 Yuanmingyuan West Road, Beijing 100193, China.; c Public Order Unit of Beijing Municipal Public Security Bureau, Beijing, China.; d Beijing Chaoyang District Center for Disease Control and Prevention, Beijing, China.; e Chaoyang District Center for Animal and Plant Disease Prevention and Control, Beijing, China.

## Abstract

**Objective:**

To evaluate the operation and outcomes of an integrated dog-mediated rabies elimination programme in the Beijing municipality, China.

**Methods:**

Beginning in 2014, the Beijing Animal Disease Control Center launched a dog-mediated rabies elimination programme in collaboration with local government and nongovernmental organizations. The programme involved: (i) a compulsory canine rabies vaccination campaign; (ii) rabies surveillance of local dog populations; (iii) educational rabies awareness programmes in public areas and schools; and (iv) the establishment of an online service platform to strengthen communications on rabies with the public.

**Findings:**

By 2023, 664 canine rabies vaccination sites had been established in the Beijing municipality, which comprises seven urban districts and nine districts with rural areas. The proportion of dogs with rabies antibodies increased from 64.7% (1115/1723) in 2014, before the programme, to 86.4% (1481/1715) in 2017 and stayed around 80% in subsequent years. In 2022, for the first time, no rabies was reported in dogs that injured people. Concurrently, the annual number of reported human rabies cases dropped from 11 in 2015 to zero in 2021, with no subsequent cases reported up until the third quarter of 2024.

**Conclusion:**

The rabies elimination programme met the goal of eliminating human rabies infections in the Beijing municipality and demonstrated that dog-mediated rabies elimination is achievable at the provincial level. The experience gained could serve as a practical guide for dog-mediated rabies control in both urban and rural areas of China and in other countries facing similar challenges.

## Résumé

**Objectif:**

Évaluer le fonctionnement et les résultats d’un programme intégré d’élimination de la rage d’origine canine dans la municipalité de Pékin en Chine.

**Méthodes:**

À partir de 2014, le Centre de contrôle des maladies animales de Pékin a lancé un programme d’élimination de la rage d’origine canine, en collaboration avec les autorités locales et des organisations non gouvernementales. Le programme comprenait les points suivants: (i) une campagne de vaccination obligatoire contre la rage canine; (ii) la surveillance de la rage parmi les populations canines locales; (iii) des programmes éducatifs de sensibilisation au sujet de la rage dans les lieux publics et les écoles; et (iv) la mise en place d’une plateforme de services en ligne destinés à renforcer la communication sur la rage avec le public.

**Résultats:**

En 2023, 664 sites de vaccination contre la rage canine avaient été établis dans la municipalité de Pékin, qui comprend sept districts urbains et neuf districts ruraux. La proportion de chiens porteurs d’anticorps antirabiques est passée, avant le programme, de 64,7% (1115/1723) en 2014 à 86,4% (1481/1715) en 2017 et s’est maintenue autour de 80% les années suivantes. En 2022, pour la première fois, aucun cas de rage n’a été signalé chez des chiens ayant mordu des personnes. Parallèlement, le nombre annuel de cas de rage humaine déclarés a chuté de 11 en 2015 à zéro en 2021, et aucun autre cas n’a été déclaré jusqu’au troisième trimestre de 2024.

**Conclusion:**

Le programme d’élimination de la rage a atteint l’objectif d’élimination des infections de rage humaine dans la municipalité de Pékin et a démontré que l’élimination de la rage d’origine canine est réalisable à l’échelle provinciale. L’expérience acquise pourrait servir de guide pratique pour réguler la rage d’origine canine dans les zones urbaines et rurales de Chine et dans d’autres pays confrontés à des défis similaires.

## Resumen

**Objetivo:**

Evaluar la implementación y los resultados de un programa integral para la eliminación de la rabia mediada por perros en el municipio de Pekín, China.

**Métodos:**

Desde 2014, el Centro de Control de Enfermedades Animales de Pekín puso en marcha un programa para la eliminación de la rabia mediada por perros, en colaboración con el gobierno local y organizaciones no gubernamentales. El programa incluyó: (I) una campaña obligatoria de vacunación antirrábica canina; (ii) la vigilancia epidemiológica de la rabia en poblaciones locales de perros; (iii) programas educativos de concienciación sobre la rabia en espacios públicos y escuelas; y (iv) el establecimiento de una plataforma de servicios en línea para fortalecer la comunicación con la población sobre la rabia.

**Resultados:**

Para 2023, se habían establecido 664 puntos de vacunación antirrábica canina en el municipio de Pekín, que comprende siete distritos urbanos y nueve distritos con áreas rurales. El porcentaje de perros con anticuerpos contra la rabia aumentó del 64,7% (1115/1723) en 2014, antes del inicio del programa, al 86,4% (1481/1715) en 2017 y se mantuvo alrededor del 80% en los años posteriores. En 2022, por primera vez, no se notificaron casos de rabia en perros que hubieran causado lesiones a personas. Paralelamente, el número anual de casos de rabia humana notificados descendió de 11 en 2015 a cero en 2021, sin que se hayan reportado nuevos casos hasta el tercer trimestre de 2024.

**Conclusión:**

El programa de eliminación de la rabia logró el objetivo de erradicar las infecciones por rabia en humanos en el municipio de Pekín y demostró que la eliminación de la rabia mediada por perros es viable a nivel provincial. La experiencia adquirida podría servir como referencia práctica para el control de la rabia mediada por perros tanto en zonas urbanas como rurales de China y en otros países que enfrentan desafíos similares.

## ملخص

الغرض تقييم طريقة تشغيل ونتائج البرنامج المتكامل للقضاء على السُعار المنقول بواسطة الكلاب، في بلدية بكين، الصين.

الطريقة بدءًا من عام 2014، أطلق مركز مكافحة الأمراض الحيوانية في بكين برنامجًا للقضاء على السُعار المنقول بواسطة الكلاب، بالتعاون مع الحكومة المحلية والمنظمات غير الحكومية. وشمل البرنامج: (أ) حملة تطعيم إلزامية ضد سُعار الكلاب؛ و(ب) مراقبة السُعار لمجموعات الكلاب المحلية؛ و(ج) برامج تعليمية للتوعية بالسُعار في الأماكن العامة والمدارس؛ و(د) إنشاء منصة للخدمة عبر الإنترنت لتعزيز التواصل مع الجمهور حول السُعار.

النتائج في عام 2023، تم إنشاء 664 موقعًا لتطعيم الكلاب ضد السُعار في بلدية بكين، والتي تضم سبع مناطق حضرية وتسع مناطق ريفية. ارتفعت نسبة الكلاب التي تحمل أجسامًا مضادة للسُعار من %64.7 (1115/1723) في عام 2014 قبل البرنامج، إلى %86.4 (1481/1715) في عام 2017، وظلت عند حوالي %80 في السنوات اللاحقة. في عام 2022، ولأول مرة، لم يتم الإبلاغ عن أي إصابة بالسُعار في الكلاب التي أصابت الأشخاص. وفي الوقت نفسه، انخفض العدد السنوي لحالات السُعار البشرية المبلغ عنها من 11 حالة في عام 2015، إلى صفر في عام 2021، مع عدم الإبلاغ عن أي حالات لاحقة حتى الربع الثالث من عام 2024.

الاستنتاج حقق برنامج القضاء على السُعار هدف القضاء على إصابات السُعار البشرية في بلدية بكين، وأثبت أنه من الممكن القضاء على سُعار الكلب المنقول بواسطة الكلاب على مستوى المقاطعات. إن الخبرة المكتسبة يمكن أن تكون بمثابة دليل عملي لمكافحة السُعار المنقول بواسطة الكلاب في كل من المناطق الحضرية والريفية في الصين، وفي الدول الأخرى التي تواجه تحديات مماثلة.

## 摘要

**目的:**

评估中国北京市犬类传播狂犬病综合消除计划的实施和成果。

**方法:**

2014年以来，北京市动物疫病预防控制中心与当地政府机构和非政府组织合作开展了一项犬类传播狂犬病消除计划。该计划包括：(i) 强制性犬类狂犬病疫苗接种活动；(ii) 对当地犬群进行狂犬病监测；(iii) 在公共场所和学校开展狂犬病宣传教育活动；(iv) 建立在线服务平台，就狂犬病相关信息加强与市民的沟通交流。

**结果:**

截至2023年，北京市已建立664个犬类狂犬病疫苗接种点，覆盖七个城市地区和九个涉农地区。获得狂犬病抗体犬群比例从2014年的64.7%（1,115/1,723）升高至2017年的86.4%（1,481/1,715），并在随后几年均保持在80%左右。2022年首次实现全年无伤人犬感染狂犬病病例报告。与此同时，每年报告的人间狂犬病病例数从2015年的11例下降至2021年的0例，并且直至2024年第三季度均未报告后续病例。

**结论:**

狂犬病消除计划实现了在北京市消除人间狂犬病病例的目标，并且表明在省级层面消除犬类传播狂犬病可以实现。所获经验可为中国城市和农村地区以及面临类似挑战的其他国家所借鉴，为控制犬类传播狂犬病提供实用指南。

## Резюме

**Цель:**

Оценить работу и результаты комплексной программы по ликвидации бешенства среди собак в муниципалитете Пекина, Китай.

**Методы:**

С 2014 года Пекинский центр по борьбе с болезнями животных в сотрудничестве с местными властями и неправительственными организациями начал программу по ликвидации бешенства среди собак. Программа включала: (i) кампанию по обязательной вакцинации собак от бешенства; (ii) наблюдение за местной популяцией собак; (iii) образовательные программы для повышения осведомленности о бешенстве в общественных местах и школах; (iv) создание платформы онлайн-услуг для укрепления связи с населением по вопросам бешенства.

**Результаты:**

К 2023 году в муниципалитете Пекина, который включает семь городских округов и девять округов с сельской местностью, было организовано 664 пункта вакцинации собак против бешенства. Доля собак с антителами к бешенству увеличилась с 64,7% (1115/1723) в 2014 году, до начала программы, до 86,4% (1481/1715) в 2017 году и оставалась около 80% в последующие годы. В 2022 году впервые не было зарегистрировано ни одного случая бешенства у собак, ранивших людей. Одновременно с этим ежегодное число зарегистрированных случаев бешенства среди людей снизилось с 11 в 2015 году до нуля в 2021 году, и в дальнейшем не было зарегистрировано ни одного случая вплоть до третьего квартала 2024 года.

**Вывод:**

Программа по ликвидации бешенства достигла цели по устранению случаев заражения людей бешенством в муниципалитете Пекина и продемонстрировала, что ликвидация бешенства среди собак достижима на областном уровне. Полученный опыт может послужить практическим руководством для борьбы с бешенством среди собак как в городских и сельских районах Китая, так и в других странах, сталкивающихся с аналогичными проблемами.

## Introduction

Rabies continues to pose a considerable public health challenge. After dog-mediated rabies was eliminated in Japan, countries in the World Health Organization (WHO) Region of the Americas and the European Region, the majority of human rabies cases worldwide have occurred in most countries in the WHO African, South-East Asian and Western Pacific Regions.^1^^,^^2^ The primary route of rabies transmission to humans in these regions is the bite of an infected dog.^3^^,^^4^ Strategies to interrupt dog-to-dog and dog-to-human rabies transmission are essential for achieving rabies elimination in these regions.

Although pre-exposure and post-exposure prophylaxis are effective for preventing human deaths from rabies, they do not provide an obvious approach to eliminating zoonotic rabies because they apparently have little effect on canine virus reservoirs.^5^ To eliminate rabies, therefore, it is crucial to strengthen strategies that target the source of infection. WHO, the United Nations Food and Agriculture Organization and the World Organisation for Animal Health have all emphasized that a One Health approach involving effective intersectoral cooperation is the mainstay of strategies for eliminating dog-mediated human rabies.^2^^,^^6^^,^^7^ Implementation of these strategies has helped many countries mitigate the burden of rabies or maintain elimination.^8^^,^^9^ However, limited empirical evidence is available on the development of a sustainable rabies elimination programme at the provincial level in a middle-income country that has a complicated geographical and cultural environment containing large human and dog populations.

China is grappling with one of the most severe rabies burdens globally: the country ranks second in terms of rabies incidence and rabies is the third leading cause of notifiable disease within the country.^10^ As in most countries with a heavy rabies burden, dogs are the principal disease reservoirs and vectors of rabies transmission.^11^^,^^12^ In Beijing, the capital of China, rapid growth in the dog population since the beginning of the 21st century has posed an unprecedented challenge for dog-mediated rabies control.^13^^,^^14^ For 11 years from 1994 to 2005, no human cases of rabies virus infection were reported in Beijing. However, between 2005 and 2013, a total of 48 human rabies cases occurred, with the annual number peaking at 13 cases in 2012.^14^ This surge highlighted a rising trend in rabies incidence. All these human cases were attributed to dog-mediated transmission, underscoring the importance of eliminating dog-mediated rabies for the prevention and control of human cases. Although the mass vaccination of dog populations has been recognized for over a century as effective for preventing viral transmission to humans,^2^^,^^15^ dogs have not been subject to mandatory rabies vaccination during much of this period in China. In addition, the lack of comprehensive rabies surveillance in dog populations and people’s inadequate knowledge of rabies prevention have also impeded progress in prevention and control.

To combat and eliminate dog-mediated rabies in Beijing, a series of integrated tactics has been implemented over the last decade, including mass canine vaccination, intensified dog rabies surveillance, rabies education, and online data management and rabies services. In recent years, Beijing has eliminated dog-mediated human rabies thanks to the collaboration of local government entities and nongovernmental organizations (NGOs). The aim of this study was to review the rabies elimination strategies used in Beijing to guide further expansion of the rabies elimination programme in China, and to assist other countries struggling with comparable challenges.

## Methods

### Study design and setting

**Fig. 1:**
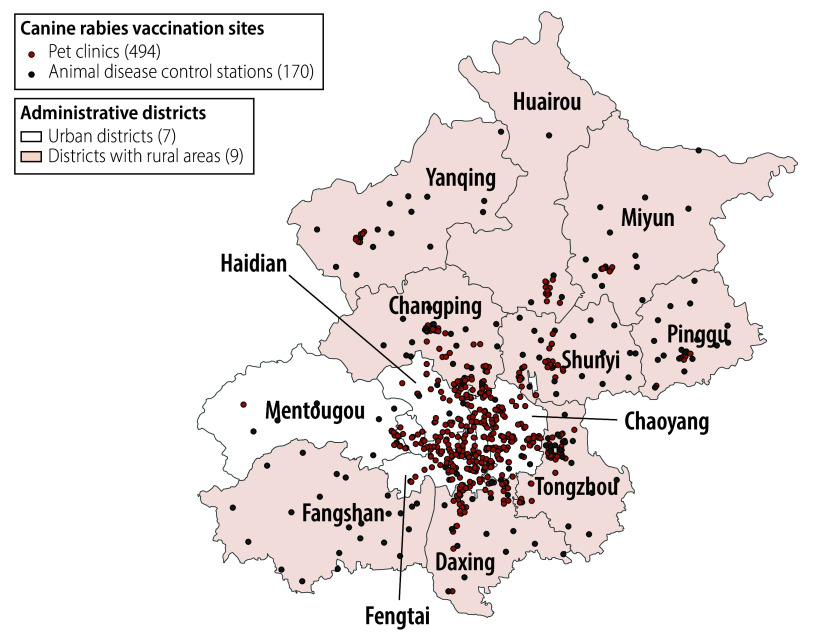
Administrative districts and canine rabies vaccination sites, Beijing municipality, China, 2014–2024

We performed a retrospective observational study of the operation and outcomes of a dog-mediated rabies elimination programme in the Beijing municipality, which comprises seven urban districts and nine districts with rural areas (Fig. 1), covers a total area of 16 410 km^2^ and has a resident population of nearly 22 million.^16^

In 1995, the number of registered dogs in Beijing was 96 000. By 2009, this figure had increased nearly 10-fold to 900 000 and has remained stable in subsequent years.^17^ Historically, before implementation of the programme, the rabies immunization status of local dog populations was unclear. In people, an 11-year streak of no reported human rabies cases was interrupted in 2005 and, subsequently, the incidence showed an upward trend.

### Programme implementation

To eradicate dog-mediated rabies, local animal health authorities adopted a range of measures. First, a compulsory vaccination campaign targeting the canine population was launched. This initiative was complemented by the sequential introduction of various government directives, including the Rabies Compulsory Immunization Management Measures; the Canine Immunization Identification Management Measures; and the Rabies Immunization Technical Training and Assessment Plan.^18^ These instruments were intended to guarantee the smooth execution of the obligatory vaccination programme. Dogs aged 3 months and older were required to receive their initial rabies vaccination as soon as possible, followed by annual booster shots. Vaccination was free for dogs that were registered and inspected annually. In addition, dog owners were exempted from paying immunization fees during regional and emergency vaccination activities.

Since its launch in October 2014, the canine vaccination campaign has been implemented continuously. The entities involved in vaccination differed according to local geographical and cultural conditions, as well as in response to the current status of the local canine population. In urban areas, the initiative was mainly anchored in pet clinics, whereas in suburban areas it involved collaborations between pet clinics and village-level vaccinators. In rural areas, the emphasis was on door-to-door vaccination by village-level epidemic prevention personnel. In addition to administering rabies vaccines, the personnel involved entered relevant data into online management systems, which enhanced digital oversight of rabies eradication efforts.

The second measure was rabies surveillance of the dog population by monitoring the acquisition of rabies virus antibodies and by tracking infections. Local animal health departments developed an annual serological surveillance plan for each district, which involved quarterly surveillance at urban pet clinics and rural farms and backyards. Each quarter, at least three monitoring sites were randomly chosen in both urban and rural settings and a minimum of 10 serum samples were collected at each site. We used commercial, rabies virus antibody, enzyme-linked immunosorbent assay (ELISA) kits (TianTech Institute of Biotechnology Co. Ltd, Zhuhai, China) for serology tests. These kits demonstrated 94% agreement with the gold-standard fluorescent antibody virus neutralization test for detecting rabies antibodies (Beijing TianTech Co. Ltd, unpublished data, 2024).^19^ In addition, they had a specificity of 98% and a sensitivity of 94% compared with the Bio-Rad ELISA kit (Bio-Rad Laboratories, Inc., Marnes-la-Coquette, France) that has been certified by the World Organisation for Animal Health (Beijing TianTech Co. Ltd, unpublished data, 2024).^20^

Specialized agencies with many years’ professional experience were entrusted to capture stray dogs throughout the entire municipality. Stray dogs were defined as dogs that were roaming free without identification tags or whose owners could not be identified or contacted. For virological monitoring, public security officers placed stray or aggressive dogs captured by the specialized agencies in shelters and isolated them for 10 days, during which their health was assessed daily. Any dog that had died underwent immediate diagnostic testing and was disposed of in accordance with biosafety regulations by the local animal health authority. Dogs suspected of having rabies also underwent diagnostic testing and, if the test was positive, were euthanized and disposed of in the same way. Commercial rabies immunofluorescence antigen detection kits (National Reference Laboratory for Animal Rabies, Changchun, China) were employed for virus detection in brain samples. The detection limit of this kit was 10^3^ LD_50_ per 0.03 mL, with no cross-reactivity observed for the canine distemper virus.

The third measure was an educational initiative to raise public awareness of rabies, which was titled “Joining hands to make rabies history”. To increase public awareness of rabies prevention, educational videos were displayed on electronic screens in public spaces, and education was provided onsite by service personnel. Schools joined in the educational initiative by encouraging students to embrace responsible and civilized dog ownership through interactive sessions that paired younger and older participants, thereby fostering a culture in which rabies prevention was advocated by children and their families. In addition, rabies vaccination mandatory notification forms and educational brochures on rabies prevention and control were disseminated at rabies vaccination points.

The fourth measure was the establishment of an online service platform to strengthen communication between government and the public. The Beijing Animal Disease Control Center and the Beijing Radio and Television Station collaborated to create the Beijing Urban Animal Smart Service Platform on 28 September 2022, coinciding with the 16th World Rabies Day. The platform relied on the media convergence platform of the Beijing Radio and Television Station. With the convenience and benefit of the public as its guiding principles, the online platform provides integrated access to vaccination services and quarantine management for urban animals. In addition, there is a range of user-friendly services, including: (i) information on vaccine schedules; (ii) guidance on locating canine vaccination sites; (iii) an online booking system for rabies vaccination; (iv) a tool for generating electronic immunization certificates; (v) assistance with searching for lost pets; and (vi) the dissemination of official announcements and scientific knowledge.

### Data analysis

To evaluate the implementation of the programme, we examined the number of vaccination stations established and the number of dogs vaccinated during the programme. In addition, we calculated the percentage of the dog population that had acquired rabies virus antibodies (through vaccination) using IBM SPSS Statistics version 29 (IBM, Armonk, United States of America). We also determined the number of stray dogs caught and the frequency of public education activities. To evaluate the effectiveness of the programme for eliminating human rabies, we compared the number of human cases before and during the programme.

The total number of vaccination sites established throughout the Beijing municipality was determined using data reported by district animal health authorities. Local animal disease control centres provided annual data on the number of dogs vaccinated and the proportion of dogs that had acquired antibodies. Data on the capture and disposal of stray dogs were obtained by reviewing local police records, and information on public educational activities were documented by outreach service personnel. The Beijing Center for Disease Control and Prevention recorded the annual incidence of human rabies cases. Data on the cost of the programme were compiled retrospectively by animal health and public health departments.

## Results

### Programme implementation

By 2023, 664 canine vaccination stations had been established in the Beijing municipality: 494 were in pet clinics in urban zones and 170 were in animal disease control stations in rural areas (Fig. 1). Between 2015 and 2023, the number of dogs vaccinated against rabies ranged from 471 400 to 672 690 per annum (Table 1). Since 2014, local police authorities sheltered and monitored the health status of approximately 1000 dogs each year. All suspected rabid dogs housed in public shelters underwent diagnostic testing, with approximately 100 samples tested annually.

**Table 1:** Rabies vaccinations and antibody tests in dogs, canine vaccination programme, Beijing municipality, China, 2014–2024

Year	No. dogs vaccinated against rabies	No. dogs that underwent serological tests for rabies antibodies	No. of dogs that had acquired rabies antibodies	% of dogs tested that had acquired rabies antibodies (95% CI)
2014^a^	NA	1723	1115	64.7 (62.4–67.0)
2015	628 822	1738	1260	72.5 (70.3–74.6)
2016	672 690	1756	1331	75.8 (73.7–77.8)
2017	664 283	1715	1481	86.4 (84.6–87.9)
2018	632 486	1310	1170	89.3 (87.5–90.9)
2019	615 278	1822	1328	72.9 (70.8–74.9)
2020	580 471	1890	1571	83.1 (81.4–84.8)
2021	594 516	1890	1595	84.4 (82.7–86.0)
2022	527 668	2058	1669	81.1 (79.3–82.8)
2023	471 400	1942	1603	82.5 (80.8–84.2)
2024^b^	NA	1501	1352	90.1 (88.4–91.5)

CI: confidence interval; NA: not available.

^a^ The mass dog vaccination programme was launched in October 2014.

^b^ The data presented are for the period up to and including the third quarter of 2024.

Note: Acquired rabies antibodies are assumed to be a proof of vaccination.

During the programme, there were 706 public educational events involving publicity, education and consultations, which engaged about 460 000 people across communities and villages. In addition, there were 337 educational sessions in schools involving over 8000 children. Furthermore, 2 895 791 standard rabies vaccination mandatory notification forms and 20 000 educational brochures on rabies prevention and control were distributed at vaccination sites, further supporting the programme’s outreach and educational objectives.

### Health outcomes

**Fig. 2:**
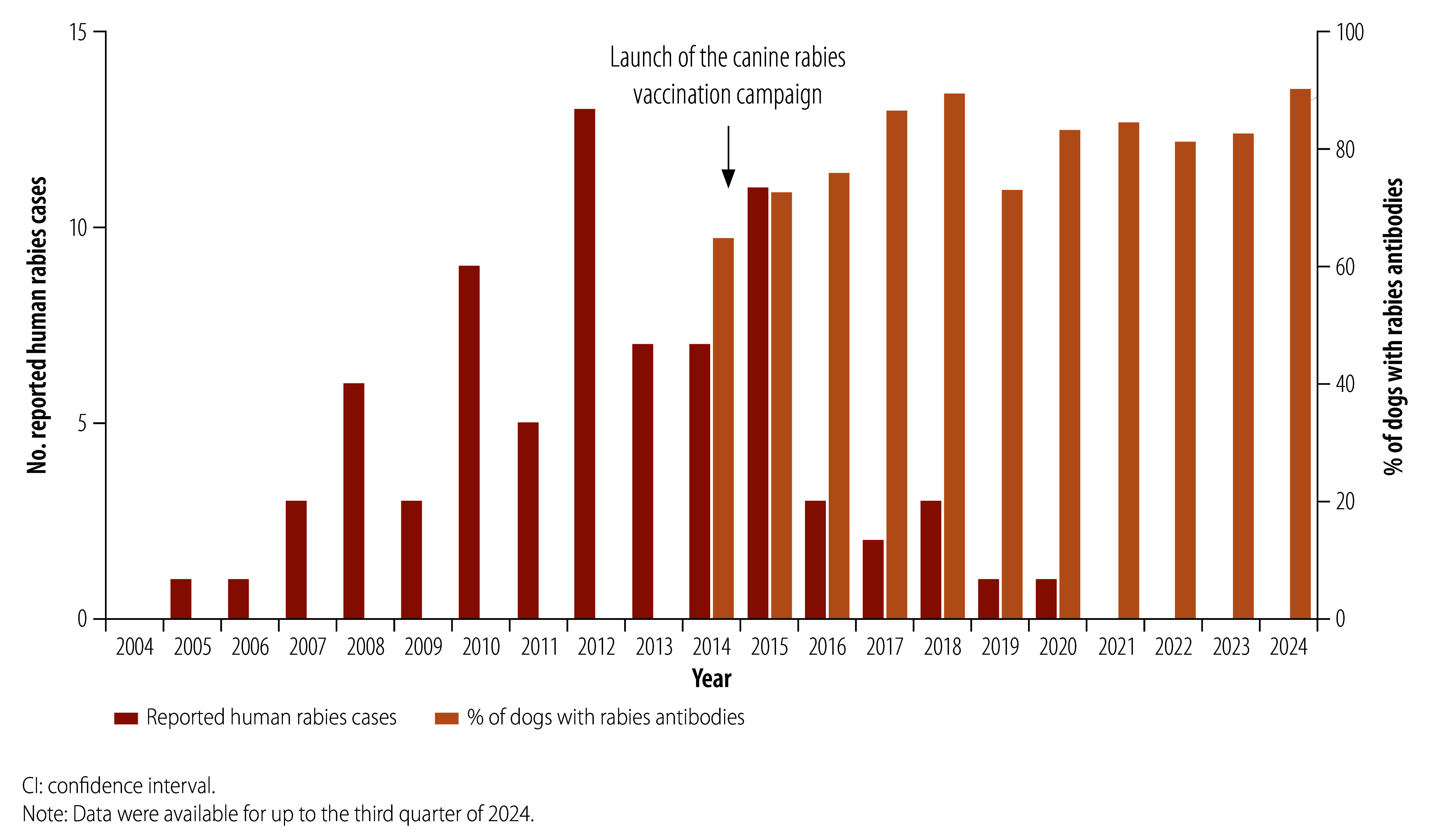
Reported human rabies cases and dogs with rabies antibodies, Beijing municipality, China, 2004 to September 2024

The proportion of dogs that had acquired rabies antibodies increased from 64.7% (1115/1723) in 2014 to 86.4% (1481/1715) in 2017, consistently remaining around 80% in subsequent years. This increase led to a steady decline in rabies incidents involving dogs implicated in human injuries, and resulted in no rabies cases being reported in these animals for the first time in 2022 (Fig. 2).

CI: confidence interval.

Note: Data were available for up to the third quarter of 2024.

In terms of public health, the peak in human rabies cases observed in 2015 (Fig. 2) was probably due to the time lag between the implementation of mass canine vaccination and the achievement of herd immunity in the dog population. The number of locally acquired human rabies cases dropped from 11 in 2015 to three in 2016, reaching the milestone of zero cases by 2021. Furthermore, no human rabies cases were reported between 2021 and the third quarter of 2024, which indicates a much lower incidence than the national average for the corresponding period (that is, approximately 0.1 cases per million people per year).^21^

### Costs

In the canine vaccination campaign, the direct cost per vaccinated dog, including expenditure on the vaccine, consumables and the administration of injections, ranged from 7 to 10.5 United States dollars (US$), primarily depending on the choice of domestic or imported vaccines. The cumulative cost of mass canine vaccination from 2015 to 2023 was approximately US$ 37.8 million, averaging US$ 4.2 million per year.

In cases of human exposure, the cost of post-exposure prophylaxis varied depending on the category of exposure.^22^ For category-II exposure, four or five vaccine injections were generally required, with each injection costing approximately US$ 14, which corresponds to a total cost of about US$ 56 to US$ 70. For category-III exposure, a rabies immunoglobulin injection, which typically cost around US$ 210, was required in addition to the rabies vaccine.

## Discussion

Over the past decade, the spread of rabies within local dog populations in the Beijing municipality has been controlled by the consistent implementation of comprehensive measures involving multiple sectors of society, such as mass dog vaccination, intensified canine rabies surveillance and public rabies education. As a result, the goal of eliminating human rabies infections was reached, with no reported cases in Beijing between 2021 and late 2024, marking a significant public health achievement.

Previously, rabies control in the WHO African Region and China has been successfully achieved in limited areas.^23^^–^^25^ Eliminating dog-mediated rabies on a provincial scale presents a diverse mix of political, technical and logistical challenges that necessitate widespread collaboration across many organizations.^26^^,^^27^ To address these challenges in Beijing, the Beijing Animal Disease Control Center spearheaded a rabies elimination programme in partnership with an array of government and NGOs, including animal health authorities, local police, public health authorities, educational institutions, village committees, animal health-care facilities and the media. Each organization played a role in targeting specific population groups and worked within a cohesive strategy. These concerted efforts were crucial for successfully eradicating both canine and human rabies.

The primary risk factor affecting rabies prevention and control in Beijing was the presence of stray dogs.^13^^,^^28^ Compared to domestic dogs, stray dogs are more susceptible to rabies virus exposure due to their life conditions and environment. Moreover, their extensive roaming patterns increase interactions with humans and other animals.^29^ Once infected with the rabies virus, stray dogs may spread the disease, thereby heightening the risk to public health. An epidemiological study conducted in Beijing found that stray dogs were responsible for 73% of dog bite incidents involving multiple victims between 2017 and 2021.^30^ In response, local animal health authorities stepped up their efforts to locate and provide shelter for stray dogs, while also improving the detection and diagnosis of rabies in unclaimed dogs, particularly those that have caused injuries to people. Local government and NGOs are collaborating to improve the management of stray dogs by developing systems to support birth control, sterilization and adoption with the aim of further minimizing the risk of rabies. 

During efforts to eliminate rabies in Beijing, communication between government bodies and the public was frequently hindered by the challenge of ensuring easy access to pertinent information.^31^ To address this challenge, local animal health authorities collaborated with reputable, influential media organizations to establish the Beijing Urban Animal Smart Service Platform. Timely dissemination of information through this digital platform facilitated the efficient management of dog populations in Beijing. Currently, this platform is expanding its function to provide online guidelines for the safe disposal of deceased pets. The platform also aims to facilitate online applications for stray animal sterilization and to promote the online adoption of abandoned animals. The goal of all these initiatives is to make the platform a convenient service point for the comprehensive management of disease in companion animals.

Implementing the rabies elimination programme across the Beijing municipality’s vast rural areas presented numerous challenges. For example, dog owners in rural areas, especially owners of older dogs, were frequently reluctant to have their dogs vaccinated. To improve the effectiveness of the programme, town authorities organized village committees that could investigate local issues involving dogs and remind dog owners to vaccinate their animals on time. In addition, animal vaccination staff provided door-to-door vaccination services in rural areas. The management of stray dogs is a recognized issue worldwide, particularly in areas where geography makes interventions more complex.^29^ To address this issue, local authorities collaborated with village committees to identify and reduce the stray dog population. Specialized capture strategies, including food baiting and multipoint encirclement, were employed to increase effectiveness in difficult terrain. The experience gained in eliminating dog-mediated rabies in Beijing using a multifaceted approach is not only applicable to urban areas but can also serve as a practical guide for rabies control in rural regions across China and other countries facing similar challenges.

Achieving and sustaining annual rabies vaccination coverage above 70% in the dog population is essential for effective rabies control and ultimate elimination.^32^^,^^33^ This level of coverage is critical for establishing herd immunity and for effectively preventing the spread of the disease.^34^ However, accurately estimating vaccination coverage in Beijing remains challenging, primarily due to a lack of reliable data, including data on the number of stray dogs. This lack of data also makes it difficult to assess the impact of stray dogs on vaccination coverage and hinders the economic evaluation of rabies control using mathematical models.^35^^,^^36^ Nevertheless, accurate quantitative evaluations of the effectiveness and efficiency of the rabies elimination programme may become achievable once further research has been conducted on enhancing management strategies for stray dogs, and on determining the characteristics of this dog population.

Following suspected rabies exposure, particularly through animal bites, the primary factor leading to human infection is inadequate post-exposure prophylaxis.^37^ Essential components of post-exposure prophylaxis include immediate and thorough wound cleaning, active immunization with rabies vaccine and passive immunization with human rabies immunoglobulin.^38^ Data collected between 2017 and 2021 indicate that, on average, 257 082 individuals in Beijing had suspected rabies exposure each year, of which 256 150 received post-exposure prophylaxis.^30^ Among people with category-III exposure, the utilization rate of rabies passive immunization agents was 52%, with some Beijing districts reporting rates less than 10%.^30^ This is a notable weak link in Beijing’s efforts to control and prevent rabies that can leave individuals at a significant risk of infection. Addressing this issue requires a comprehensive investigation of the factors influencing the uptake of passive immunization. Increased use of passive immunization will help minimize the risk of infection in the population, particularly among individuals residing in rural areas where the incidence of rabies is higher.^39^

Here we show that dog-mediated rabies elimination is achievable at the provincial level in China. The experience gained could serve as a practical guide for dog-mediated rabies control in urban and rural areas of China and in other countries facing similar challenges.
